# Amygdala Underlies the Environment Dependency of Defense Responses Induced *via* Superior Colliculus

**DOI:** 10.3389/fncir.2021.768647

**Published:** 2022-01-07

**Authors:** Kaoru Isa, Kota Tokuoka, Sakura Ikeda, Sara Karimi, Kenta Kobayashi, Thongchai Sooksawate, Tadashi Isa

**Affiliations:** ^1^Department of Neuroscience, Graduate School of Medicine, Kyoto University, Kyoto, Japan; ^2^Graduate School of Biostudies, Kyoto University, Kyoto, Japan; ^3^Physiology Research Center, Institute for Basic Sciences, Kashan University of Medical Sciences, Kashan, Iran; ^4^Section of Viral Vector Development, National Institute for Physiological Sciences, Okazaki, Japan; ^5^Department of Pharmacology and Physiology, Faculty of Pharmaceutical Sciences, Chulalongkorn University, Bangkok, Thailand; ^6^Human Brain Research Center, Graduate School of Medicine, Kyoto University, Kyoto, Japan; ^7^Institute for the Advanced Study of Human Biology (WPI-ASHBi), Kyoto University, Kyoto, Japan

**Keywords:** escape behavior, flight response, pathway-selective, optogenetics, amygdala, superior colliculus, cuneiform nucleus, mouse

## Abstract

In our previous study, we showed that the defense responses induced by the selective optogenetic activation of the uncrossed output pathway from the deeper layer of the superior colliculus were environment dependent in the mouse. In a small closed box, the stimulus frequently induced flight (fast forward run away) responses, while in a large open field, the stimulus tended to induce backward retreat responses. We tested a hypothesis that the amygdala is involved in such environment dependency of the innate defense responses. For this purpose, we made a bilateral lesion of the amygdala induced by the ibotenic acid injections in male mice. As a result, in the mice with lesions of substantial portions of the basolateral and basomedial complex, the flight responses in the closed box disappeared and retreat responses were mainly induced. The retreat responses on the open platform were unchanged. Classically, the amygdala has been considered to be involved in the memory-dependent contextual modulation of the fear responses. In contrast, the present results suggest a novel view on the role of the amygdala in which the amygdala plays a key role in sensing the current environmental setting for making a quick decision of action upon emergency, which is critical for survival in the natural environment.

## Introduction

In case you are attacked by a predator coming from above, what would you do? According to the predatory imminence theory, if the animal senses the approach of a predator, it may exhibit species-specific defense reactions such as freezing to avoid being detected by the predator (Bolles, 1970). However, once the animal is detected by its predator and a threat is imminent, freezing may not be the optimal behavior for survival. Then, the animal enters the “circa-strike” phase, where its behavior will shift from passive freezing to active flight (Fanselow and Lester, 1988). If you are in a small narrow field surrounded by the wall, moving from the center of the field to its corner and escaping along the wall would be a good strategy to protect yourself. If you are in a wide-open field where nothing hides you, simply looking above and retreating might be the best way. Thus, the strategy for you to defend yourself from the predator may depend on the environment in which you are placed. You should respond quickly at your first encounter with the predator. Otherwise, you cannot survive in this world. Thus, such behavioral responses should be innately imprinted in your nervous system. Which neural system in the brain works for you to integrate the knowledge of your current environmental settings and how your enemy is approaching, for you to make a quick decision for the best strategy to defend yourself? In other words, what is the neural basis of an innate environment-dependent decision of defense strategy?

**Figure 1:**
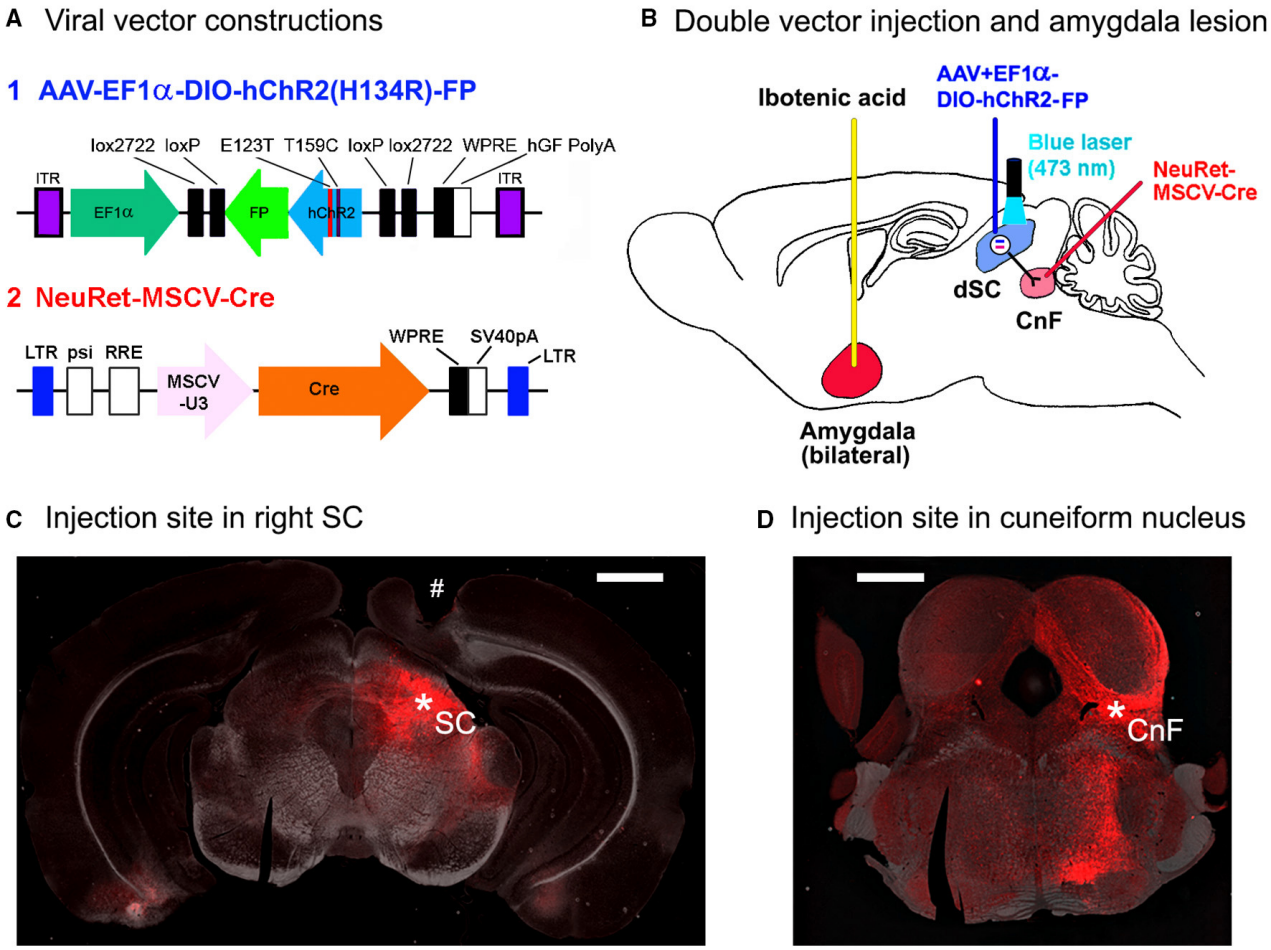
Methods for the optogenetic activation of SC defense pathway in mice. **(A)** Viral vector constructions. **(B)** The schematic diagram for the double injection of the viral vectors into the right cuneiform nucleus and the right superior colliculus (SC) and the interaction of NeuRet-MSCV-Cre and AAV-EF1α-DIO-hChR2(H134R)-FP in the double-infected SC neurons, and bilateral amygdala lesion induced by the ibotenic acid injection. **(C)** A photomicrograph of the injection site in the right SC (^*^SC). # Optic fiber tract for optogenetic stimulation. Scale bar = 1 mm. **(D)** A photomicrograph of the injection site in the right cuneiform nucleus (^*^CnF). Scale bar = 1 mm.

In our previous study, we succeeded in inducing the defense behavior in mice by selective optogenetic activation of the uncrossed output pathway from the deeper layers of the superior colliculus (dSC) (hereafter termed “superior colliculus (SC) defense pathway”) with the expression of channelrhodopsin2 (ChR2) (Isa et al., 2020). The selective expression of ChR2 was enabled by a combination of double viral vectors: injection of the anterograde vector, adeno-associated viral vector (AAV) carrying DIO-ChR2-EYFP into the dSC and injection of retrograde vector NeuRet carrying Cre into the cuneiform nucleus (CnF) ipsilateral to the dSC injection according to the classical studies by Redgrave and colleagues (Sahibzada et al., 1986; Dean et al., 1989). When we photostimulated the SC with a blue laser (473 nm wavelength), the mice showed immediate defense responses (Isa et al., 2020). Interestingly, the defense responses were dependent on the environment in which the mice were placed. If the mice were in the center of a small closed box, they frequently jumped to the wall and ran along the wall, the behavior termed “flight response” upon photostimulation of the SC. In contrast, when the mice were placed on the center of a large open platform (an elevated open circle) and received the same photostimulation to the SC, the mice tended to show a quick upward head turn, succeeded by backward locomotion, termed “retreat response.” Thus, the evoked responses were environment-dependent. Here, the flight and/or retreat were the major responses, while freezing was minor, presumably because the stimulation parameter brought the mice into the “circa-strike” phase in the predatory imminence theory (see above), where freezing may not be the optimal behavior for survival. This pattern of behavior induced by the activation of the SC defense pathway was in marked contrast to the activation of another output channel from the SC, the SC orienting pathway, which was targeted toward the contralateral medial pontomedullary reticular formation (Sahibzada et al., 1986; Dean et al., 1989; Sooksawate et al., 2013). In this case, the photostimulation of the dSC-induced contraversive head-body turn both in the closed box and open platform, which was environment independent (Isa et al., 2020). In that study, we analyzed the axonal trajectories of both pathways and found that the SC defense pathway possessed collateral projections to several nuclei in the midbrain and forebrain regions, many of which are closely related to the amygdala. It has been shown that the amygdala is involved in generating fear responses in a context-dependent manner, in which association of the information about the environment surrounding the subjects and their past experience of fear in the environment and its memory leads to fearful emotion and physiological responses, such as freezing behavior or escape, and increase in the heart rate (LeDoux et al., 1988; Johansen et al., 2011; Duvarci and Pare, 2014; Janak and Tye, 2015; Izquierdo et al., 2016; Terburg et al., 2018). We hypothesized that besides such association of the current environmental information and the past fear memory of the subject, the amygdala may play a role in sensing the current environmental setting for making a quick decision of action upon emergency, which is critical for survival in the natural environment. To demonstrate this hypothesis, we investigated the effect of amygdala lesion on the environment dependency of the defense response induced by optogenetic stimulation of the SC defense pathway (Figure 1B). The effect of the amygdala lesion on the environment dependency of the immediate defense response will be demonstrated.

## Materials and Methods

### Animals

All the experimental procedures were conducted in accordance with the Guidelines of the Ministry of Education, Culture, Sports, Science and Technology (MEXT) of Japan, reviewed and approved by the Institutional Animal Care and Use Committee of the Graduate School of Medicine, Kyoto University.

A total of 57 C57BL/6 male mice aged between 7 and 10 weeks and weighing 20–25 g were used for analysis in this study. They were housed in groups before and then individually in the transparent plastic cages after implantation of the optic fibers with free access to food and water and were maintained on a Zeitgeber time 12-h light/dark cycle. We paid careful attention to reducing the stress and also decreased the number of animals used in this study. The animal experimental protocols are shown in Table 1.

**Table 1:** Timeline for experimental protocols.

**Timeline**	**Animal procedures**
Day 0	NeuRet-MSCV-Cre lentiviral vectors injection into the right cuneiform nucleus (CnF)
3–7 days after 1st viral injection	AAV-EF1α-DIO-hChR2 (H134R)-FP injection into the right deeper layers of superior colliculus (dSC)
4–8 weeks after 2nd viral injection	Bilateral lesion of amygdala by ibotenic acid injection
3–6 days after amygdala lesion	Optic fiber implantation on the right dSC
5–31 days after optic fiber implantation	Behavior testing induced by optogenetic stimulation of the right dSC (> 6 weeks after NeuRet-MSCV-Cre lentiviral vectors injection and > 1 week after bilateral lesion of amygdala)

### Viral Vector Preparation

NeuRet-Cre lentiviral vectors were prepared as previously described (Kobayashi et al., 2016). Briefly, the packaging plasmids (pCAGkGP4.1R and pCAG4-RTR2) (Hanawa et al., 2002, 2004), envelope plasmid (pCAGGS-NeuRet) (Kato et al., 2014), and transfer plasmid (pCL20-MSCV-Cre) were transfected into HEK293T cells using the calcium phosphate method. The cultured medium was collected and filtered using a Millex-HV filter unit (0.45 μm, Merck Millipore, MA, USA). Viral vector particles were pelleted by centrifugation and suspended in phosphate-buffered saline (PBS), then an ion-exchange column chromatography using Sepharose Q FF ion-exchange column (GE Healthcare, IL, Unites States) and AKTA prime plus chromatography apparatus (GE Healthcare, IL, USA) were used to purify them. Ultrafiltration using a Vivaspin 10K MWCO filter (Vivascience AG, Hanover, Germany) was carried out to concentrate the collected fractions containing the vector particles. A Lenti-X qRT-PCR titration kit (Clontech, CA, Unites States) was employed to evaluate the copy number of the RNA genome. Real-time quantitative PCR was performed in duplicate samples using the StepOne real-time PCR system (Applied Biosystems, Foster City, CA, USA). The AAV Helper Free Expression System (Cell Biolabs, Inc., San Diego, CA, USA) was used to produce AAV vectors (Kobayashi et al., 2016). Briefly, the packaging plasmids (pAAV-DJ and pHelper) and transfer plasmid (pAAV-EF1α-double floxed-hChR2(H134R)-mCherry-WPRE-HGHpA, pAAV-EF1α-double floxed-hChR2(H134R)- EYFP-WPRE-HGHpA [Addgene, Plasmid #20297, #20298], and pAAV-CAGGS-Cre) were transfected into HEK293 T cells. A crude cell extract involving AVV vector particles was purified by ultracentrifugation with cesium chloride. The purified particles were dialyzed with PBS containing 0.001% Pluronic F-68 (Sigma-Aldrich, MO, United States) and then concentrated by using an Amicon10KMWCO filter (Merck Milli-pore, Darmstadt, Germany). The copy number of the viral genome (vg) was determined by real-time quantitative PCR using the TaqMan Universal Master Mix II (Applied Biosystems, Foster City, CA).

### Injection Surgeries

The two kinds of viral vectors were injected into the 57 mice. Anesthesia was induced by a blend of intraperitoneal (i.p.) injection of ketamine (60 mg/kg body weight) and xylazine (10 mg/kg body weight). Besides, dexamethasone (5.5 mg/kg body weight, intramuscular [i.m.]) was administered as a premedication for each surgery. The anesthetized mouse was precisely fixed on a stereotactic frame (SR-6M-HT, Narishige, Tokyo, Japan) for injections of the vectors or ibotenic acid/artificial cerebrospinal fluid (ACSF). After a small hole was made on the skull with a dental drill, microinjection was made through a glass micropipette (tip diameter, 50–70 μm) connected to a syringe pump (Legato 130; Muromachi Kikai Co, Tokyo, Japan). The injection rate was 0.1 μl/min and for efficient diffusion, the injector was kept in place for more than an extra 5 min. The coordinates for the target locations (CnF, dSC, and amygdala) were determined from the brain atlas (Franklin and Paxinos, 2008).

First, NeuRet-MSCV-Cre (Figure 1A; 0.4–0.5 μl/5 min; titer, 1.6–36 × 10^10^ copies/ml) or rAAV2 retro-CAGGS-Cre (0.5 μl/5 min; titer, 2.8–5.3 × 10^12^ vg/ml) was administered into the 2 points with different anterior-posterior coordinates with the tip-tilted 45° upward in the rostral direction in the right CnF using glass micropipette (AP: −8.2 mm, ML: 1.4 mm, DV: 3.1 mm from the surface of the cerebral cortex, and AP: −8.5 mm, ML: 1.2 mm, DV: 3.0 mm from the surface of the cerebral cortex; Figure 1D).

After 3–7 days of the first injection, AAV-EF1α-DIO-hChR2 (H134R)-EYFP/mCherry (FP) (Figure 1A; 0.5 μl/5 min; titer, 7.9–25 × 10^12^ vg/ml) was injected into the right dSC (AP: −3.8 mm, ML: 1.2 mm, DV: 1.6 mm from the surface of cortex; Figure 1C).

### Amygdala Lesion

After 4–8 weeks of the second injection of the viral vector described above, the mice were divided into two groups, sham-lesioned group (*n* = 36) and amygdala-lesioned group (*n* = 21). The former group received ACSF (concentration in mM: NaCl, 127; KCl, 1.5; KH_2_PO_4_, 1.24; MgSO_4_, 1.4; CaCl_2_, 2.4; D-glucose, 10; NaHCO_3_, 26) in the amygdala complex, while in the latter group, the lesions were produced by bilateral injection of ibotenic acid (concentration: 10 μg/μl ACSF) at the stereotaxic coordinates of AP: −1.7 mm, ML: 2.85 mm, and DV: 4.4 mm. The volume of injection was 0.3 μl on each side.

### Optic Fiber Implantation

In all the mice, 3–6 days after the amygdala lesion, the plastic optical fibers (500 μm diameter) (COME2-DF1-500, Lucir Inc., Tsukuba, Japan) were implanted just above the SC surface through the cortical tissue (AP: −3.8 mm, ML: 1.2 mm right, DV: 0.8–1.0 mm from the surface of the cerebral cortex) with the stereotaxic surgery as mentioned above. An example of the fiber track is shown with “#” in the histological section in Figure 1C.

### Behavioral Tests

After 5–31 days of the optical fiber implantation, behavioral responses to laser stimulation by blue laser (473 nm wavelength; Model COME2-OFC-1, Lucir Inc., Tsukuba, Japan) were investigated. The behavior tests in all mice were taken for more than 6 weeks after NeuRet-MSCV-Cre lentiviral or rAAV2 retro-CAGGS-Cre vectors injection and more than 1 week after bilateral lesion of the amygdala.

A closed box (16 cm wide, 25 cm long, and 31 cm high) and an open elevated circular field (open platform, 40 cm diameter and placed at 1 m height from the floor) were used to examine the environmental dependency of the effect of photostimulation of the SC. Bedding material was scattered on the closed box, but its presence never affected the behavior of the animals. Both tests were performed on each mouse. The intensity of the laser illumination through the optical fiber with a 500 μm diameter was 147–253 mW/mm^2^.

The SC defense pathway neurons were stimulated by the photostimulation with a single pulse of 50 or 200 ms duration or repetitive stimulation of 50 ms-on and 50 ms-off duration at 10 Hz frequency by 5 × or 20 × repetitions. To prevent the behavioral response adaptation by repeated stimulus, the stimulations with various parameters were applied randomly. EthoVision video tracking software (XT 15, Noldus Information Technology, the Netherlands) was used to analyze behavioral data.

Behavioral responses were classified into retreat, flight, and others. A quick upward-directed head-only turn was frequently followed by the backward movements (retreat) and/or fast forward run away (flight). In such cases, they were categorized by these flight or retreat responses that succeeded after head-only turns. “Others” included head-only turns, body turn, or freezing, but as shown in **Figure 3**, they were relatively minor compared with the retreat or flight responses in the dataset of this article.

### Histological Assessments

After finishing the behavioral tests, the mice were deeply anesthetized with sodium pentobarbital (80 mg/kg body weight, i.p.). After confirming the disappearance of reflexive responses, transcardial perfusion was conducted with 0.05 M PBS followed by 4% paraformaldehyde in 0.1 M phosphate buffer (pH 7.4). Then, the brain and spinal cord were removed, and successively postfixed, cryoprotected, and coronal sections of 40 μm thickness were prepared using a sliding microtome (Retoratome REM-710, Yamato, Asaka, Japan).

The direct fluorescence of specimens after anti-FP immunohistochemistry (IHC) was observed to confirm the expression of ChR2 in the double-infected SC defense pathway neurons.

For IHC against EYFP, a rabbit polyclonal antibody to GFP (AB_221569, ThermoFisher #A11122, MA, USA) was used. The sections were incubated with the anti-GFP antibody (1:2,000–5,000, in the 0.3% Triton-X in phosphate buffer solution (PBS-T) for 16 h at 4°C after blocking incubation with PBS-T containing 5% normal goat serum (NGS) (Vector Laboratories, USA, #S-1000) at room temperature. The sections were then washed in PBS-T and incubated with AlexaFluor 594-conjugated anti-rabbit IgG (1:200; Life Technologies, Japan) in PBS-T.

To obtain the permanent staining of the SC defense pathway neurons in the present experiments, DAB staining was conducted (data not shown) (Isa et al., 2020). Briefly, the sections were incubated in 0.6% H_2_O_2_ in Dent's fixative followed by blocking with 5% skimmed milk. Next, the sections were incubated in the anti-GFP antibody (1:5,000) overnight at 4°C. The next day, the sections were incubated in biotin goat anti-rabbit IgG (1:200; Vector Laboratories, USA, # BA-1000) in PBS-T for 2 h, and afterward, the reaction in the Vectastain Elite ABC kit (1:200; Vector laboratories, CA, USA) was induced. DAB (1:10,000; Wako, Japan) containing 1% nickel ammonium sulfate and 0.0003% H_2_O_2_ in Tris-buffered saline was used to visualize the labeled neurons.

For IHC against mCherry, a rabbit polyclonal antibody to RFP (Rockland Immunochemicals, Gilberstville, PA, USA) was used. Sections were incubated in a blocking solution of 10% NGS in PBS with 0.3% of Triton X-100 (PBS-T). Sections were then incubated in the anti-RFP in 2% NGS/PBS-T solution overnight at 4°C. On the next day, sections were washed in PBS-T and incubated in 1:200 AF488-conjugated goat anti-rabbit IgG (Invitrogen, MA, USA) for 2 h or 1:200 biotinylated goat anti-rabbit IgG (Vector Laboratories, CA, USA) in 2% NGS/PBS-T for 2 h followed by Vectastain Elite ABC kit and DAB staining as described above.

Nissl and anti-NeuN immunostainings were performed for recognizing the amygdala lesion induced by the ibotenic acid injection. First, the Nissl-stained sections were carried out to roughly identify the location of the damaged area (data not shown).

The exact location of the damage caused by ibotenic acid was successively estimated by anti-NeuN immunostaining. Anti-NeuN immunostaining was visualized with DAB as a chromogen. Here, the rinsed sections were incubated in a solution of 0.6% H_2_O_2_ in Dent's fixative followed by incubation in a blocking solution of 10% NGS in PBS-T for 1 h at room temperature. Then, the sections were incubated in a mouse monoclonal anti-NeuN antibody (close A60, 1:400; Millipore, # MAB377) in PBS-T/NGS at 4°C overnight. On the second day, the sections were rinsed four times in PBS-T. Then, they were incubated for 2 h with biotin goat anti-mouse IgG (1:200; Vector Laboratories, USA, #BA9200) in PBS-T/NGS and at room temperature. Next, after washing four times in PBS-T, the sections were incubated in ABC-Elite (1:200; Vector laboratories, USA) solution in PBS-T for 1 h. After washing the sections with PBS, they were progressed to the process of DAB staining as mentioned above.

A light microscope (BZ-X710 and BZ-X810, Keyence, Osaka, Japan) was utilized for observing and capturing the images of the visualized neurons and fibers.

### Statistical Analysis

Data are expressed as mean ± SD or SEM. Significance was tested using one-way ANOVA and Bonferroni's multiple comparison test, where applicable, and *p* < 0.05 was considered to be significant.

## Results

Among the 57 mice, including 36 sham-lesioned mice and 21 mice with amygdala lesion induced by the ibotenic acid injection, 11 sham-lesioned mice and 10 mice with bilateral amygdala lesion exhibited successful expression of ChR2 throughout the SC defense pathway (detailed in Isa et al., 2020) with vigorous motor responses to the optogenetic stimulation, and successful accomplishment of all the experimental procedures were initially selected for further analysis. However, we excluded two mice with bilateral amygdala lesion later because the lesion extent in these animals was small and covered only a part of the amygdala according to the anti-NeuN immunostaining, as described below.

### Extent of Amygdala Lesions

**Figure 2:**
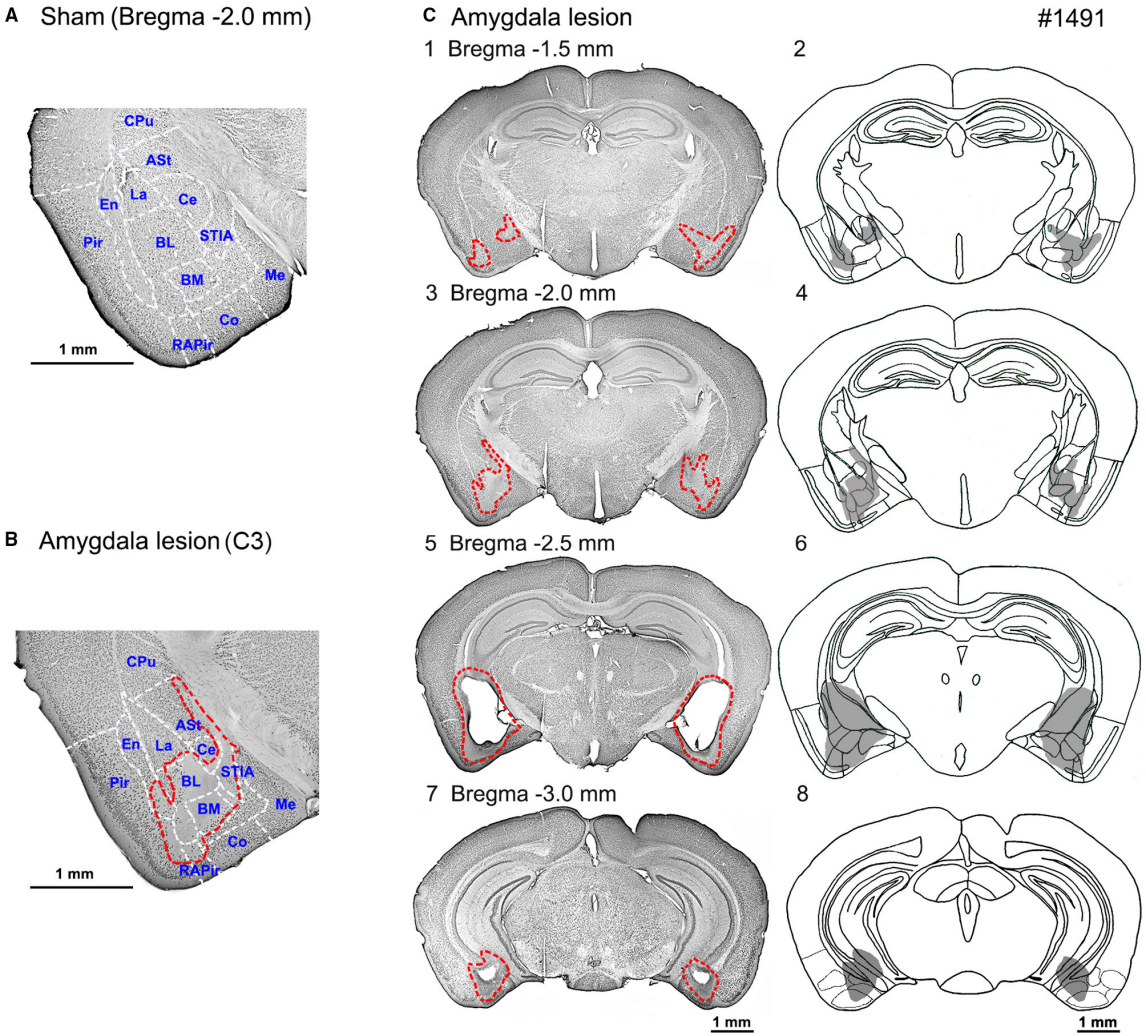
Photomicrographs and schematic diagrams of the frontal sections of mouse brains showing lesion extents in bilateral amygdala induced by the ibotenic acid injections. **(A)** A photomicrograph of the frontal section of amygdala slice (Bregma −2.0 mm) in a sham-lesioned mouse. **(B)** A photomicrograph of the frontal section of the amygdala (Bregma −2.0 mm) shows lesion extent (red dot line). **(C)** Photomicrographs (left panel; **C1, C3, C5, C7**) and schematic diagrams (right panel; **C2, C4, C6, C8**) of the serial frontal sections (Bregma −1.5 to −3.0 mm) showing the lesion extents (red dot line or gray area, respectively) in mouse No. 1491. ASt, amygdalostriatal transition area; BL, basolateral amygdaloid nucleus; BM, basomedial amygdaloid nucleus; Ce, central amydaloid nucleus; Co, cortical amygdaloid nucleus; CPu, caudate putamen (Striatum); En, endopiriform claustrum; La, lateral amygdaloid nucleus; Me, medial amygdaloid nucleus; Pir, piriform cortex; RAPir, rostral amygdalopiriform area; STIA, bed nucleus of the stria terminalis, intraamygdaloid division.

Histological assessment of the amygdala lesion was primarily conducted with anti-NeuN immunostaining and compared with the sham-lesioned mice (Figure 2 and Table 2). In all the 11 sham-lesioned mice with vigorous behavioral responses to the photostimulation, the bilateral amygdala remained intact. Figure 2 exemplifies the extent of the lesion in one case (1491). Figures 2A,B shows the sections of anti-NeuN immunostaining at the level of Bregma −2.0 mm (the level of C3 and C4) in the sham-lesioned mouse and 1491, respectively. By comparison, we could delineate the extent of the lesion as indicated with the red dashed line in Figures 2B,C. Figure 2C shows the lesion extent thus identified in four different coronal planes. Such analysis was conducted in all 10 mice administered with the ibotenic acid injection. The pseudoquantitative rankings of lesion extent (from – to +++) in and around the amygdala, including the caudate/putamen (CPu), piriform cortex (Pir), endopiriform claustrum (En), and hippocampus (Hip), are indicated in Table 2, which will be argued about the behavioral effect in the following section.

**Table 2:** Lesion extent induced by the ibotenic acid injection into the bilateral amygdala.

**Mouse no**.		**Amygdala**							**CPu**	**Pir**	**En**	**Hip**
		BL	BM	Ce	Ast	STIA	AHi	Co				
1290	Right	++	++	+	++	++	++	++	-	++	+	+
	Left	++	+	++	+	+	+++	++	-	+	+	+
1291	Right	+++	++	+	++	+++	+++	++	-	+	+	+
	Left	++	++	+	++	+++	++	+	+	+	+	+
1292	Right	-	+	-	-	+	+	-	-	-	+	+
	Left	++	++	-	-	-	-	+	-	+	-	-
1300	Right	++	+	++	+	++	-	-	+	-	-	+
	Left	++	+	-	-	-	-	+	-	+	+	+
1309	Right	++	++	+	+++	+++	+++	++	+	+	+	+
	Left	++	++	++	+++	+++	++	++	-	+	+	+
1491	Right	++	++	+	-	+++	+	++	-	+	-	+
	Left	+++	++	+	-	+++	++	++	-	+	+	+
3	Right	+	+	-	-	-	+	+	-	-	-	-
	Left	++	++	+	-	++	+	+	-	+	++	-
2	Right	++	+	++	++	+++	+	-	-	-	-	-
	Left	+++	++	++	-	++	+	+	-	+	-	-
1494*	Right	+	-	+	+	+	+	-	-	-	-	-
	Left	+	-	+	++	++	-	-	-	+	+	-
1*	Right	+	+	+	+	++	+	+	-	-	-	-
	Left	+	+	+	+	+	+	+	-	+	+	-

*+, <50% of lesion; ++, >50% of lesion; +++, 100% of lesion*.

The animals in the bottom two hatched rows (1494* and 1*) were excluded from the behavioral analysis.

*AHi, amygdalohippocampal area; ASt, amygdalostriatal transition area; BL, basolateral amygdaloid nucleus; BM, basomedial amygdaloid nucleus; Ce, central amydaloid nucleus; Co, cortical amygdaloid nucleus; CPu, caudate putamen (Striatum); En, endopiriform claustrum; Hip, hippocampus; Pir, piriform cortex; STIA, bed nucleus of the stria terminalis, intraamygdaloid division*.

### Effects of Amygdala Lesion on the Environment Dependency of Defense Responses

**Figure 3:**
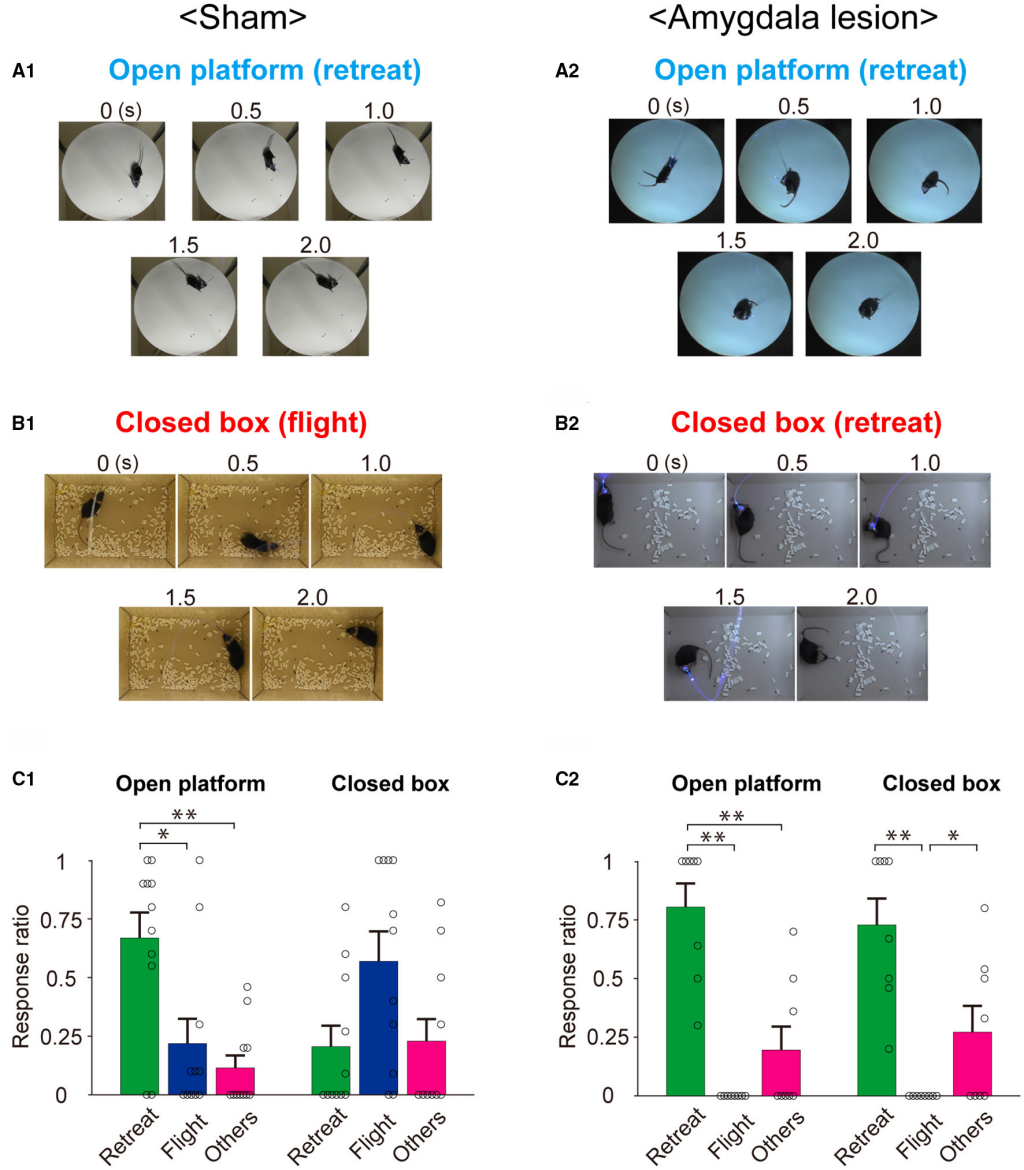
Behavioral responses to optogenetic activation of the SC defense pathway in sham-lesioned and amygdala-lesioned mice. **(A,B)** Sequential photographs illustrating typical environment-dependent behavioral responses following the optogenetic activation of the SC defense pathway on the open platform **(A)** and in the closed box **(B)** in the sham-lesioned **(A1,B1)** and amygdala-lesioned mice **(A2,B2)**. **(C)** Ratios of individual response patterns induced by the optogenetic activation of SC defense pathway on the open platform and in the closed box in the sham-lesioned [**(C1)**, *n* = 11] and amygdala-lesioned [**(C2)**, *n* = 8] mice (*p < 0.05, **p < 0.01, Bonferroni's multiple comparison test).

**Figure 4:**
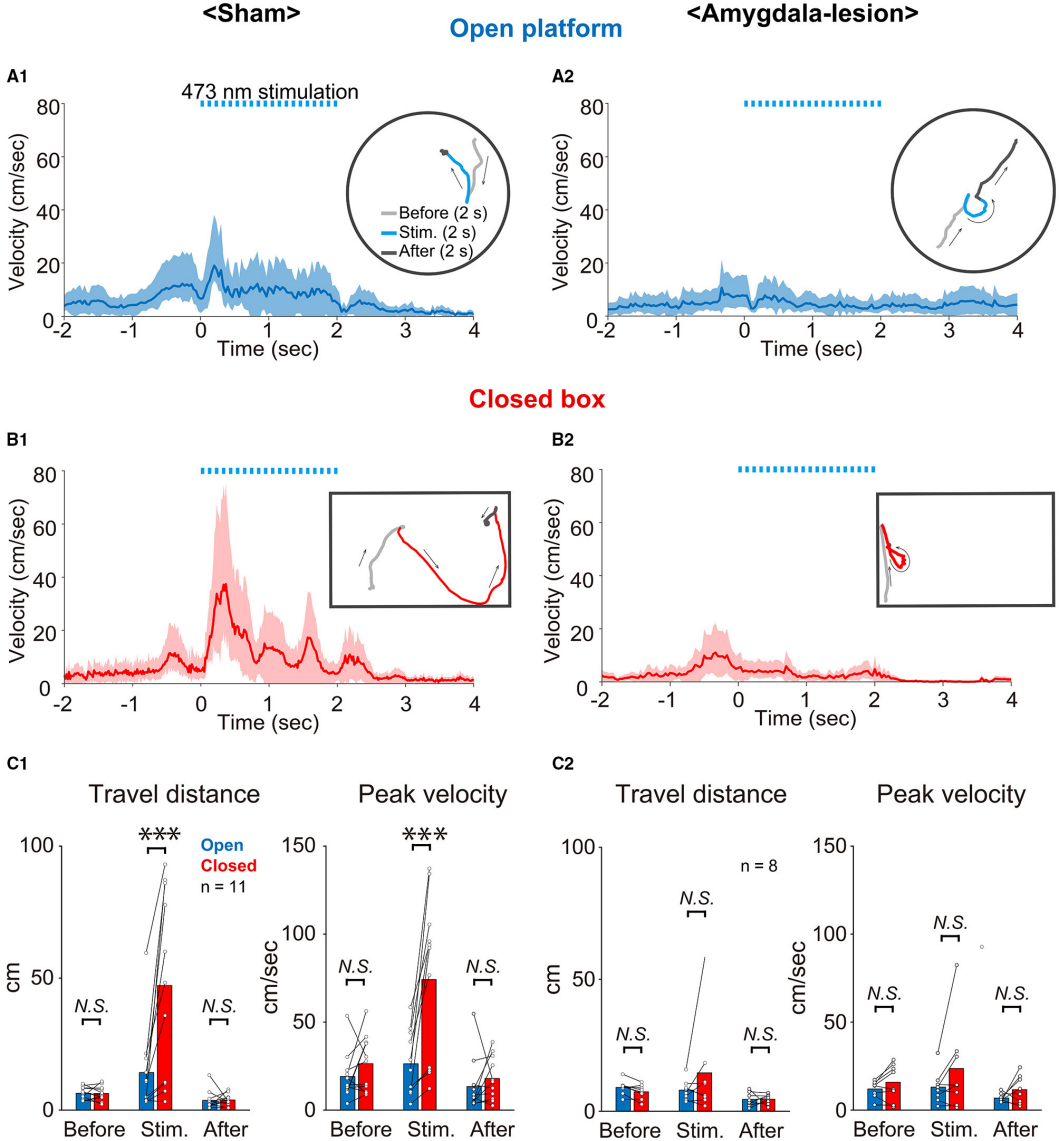
Analysis of behavioral responses. **(A,B)** Averaged time course of locomotion speed and its trajectory (inset) of an example mouse on the open platform **(A1,A2)** and in the closed box **(B1,B2)** (*n* = 10 trials in each) of sham-lesioned **(A1,B1)** and amygdala-lesioned **(A2,B2)** mice. The shaded area indicates the standard deviation of the averaged data. Dashed light-blue lines indicate 473 nm photostimulation (20 trains of 50 ms pulses at 10 Hz). **(C1,C2)** Quantitative analysis of travel distance and peak velocity before (2 s), during (2 s), and after (2 s) optogenetic activation on the open platform and in the closed box of sham-lesioned mice **(C1)** (*n* = 11 mice; ***: *p* < 0.001, *N.S.: p* > 0.05, Bonferroni's multiple comparison test) and amygdala-lesioned mice **(C2)** (*n* = 8; *N.S*.: *p* > 0.05, Bonferroni's multiple comparison test).

To clarify the role of the amygdala in the environment dependency of the defense responses induced by optogenetic activation of the SC defense pathway, the stimulus effects were compared between the sham-lesioned mice and the amygdala-lesioned mice. Figure 3A1 shows the sequential photographs of an example retreat response on the open platform, and Figure 3B1 shows those of an example flight response in the small closed box during the activation of the SC defense pathway neurons (50 ms × 20 pulses) in the same sham-lesioned mouse. The video films of the trials in both cases are presented in Supplementary Videos 1, 2, respectively. The insets in Figures 4A1,B1 show the single movement trajectories before, during, and after the retreat response shown in Figure 3A1 on the open platform and flight response shown in Figure 3B1, respectively. These were measured using the EthoVision system. Figures 4A1,B1 shows the averaged velocity profile of responses on the open platform (*n* = 10 trials in a session, all the responses were retreat) and those in the closed box (*n* = 10 trials in a session, all the responses were flight). As shown in these figures, the peak velocity was much higher for the flight than the retreat. Figure 3C1 shows the frequency of individual behavioral responses (retreat, flight, or others) in the closed box and on the open platform in the 11 sham-lesioned mice. Here, as shown in our previous report (Isa et al., 2020), environment dependency of behavioral responses was clearly detected; retreat responses were more frequently observed than the flight responses (*n* = 11; ^*^: *p* < 0.05, Bonferroni's multiple comparison test) and others (*n* = 11; ^**^: *p* < 0.01, Bonferroni's multiple comparison test) on the open platform, while flight responses were most frequently common in the closed box despite significant difference was not detected (*n* = 11; *N.S.: p* > 0.05, Bonferroni's multiple comparison test). Reflecting such behavioral response patterns, the average of the travel distance and peak velocity during the stimulation period in the closed box was longer and faster than those on the open platform (*n* = 11; ^***^: *p* < 0.001, *N.S.: p* > 0.05, Bonferroni's multiple comparison test) (Figure 4C1).

Figures 3A2,B2,C2 and Figures 4A2,B2,C2 show the results of mice with bilateral amygdala lesion. As exemplified in Figures 3A2,B2 and Supplementary Video 3, the mouse showed retreat response on the open platform as well as the sham-lesioned mice (Figure 3A1), and they showed mostly retreat responses also in the closed box (Figure 3B2 and Supplementary Video 4). As shown in the example sessions in the insets of Figures 4A2,B2, the velocity of movement responses in the closed box (Figure 4B2) was slow as those on the open platform shown in Figure 4A2. Figure 3C2 shows the frequency of individual behavioral responses (retreat, flight, or others) on the open platform and in the closed box of the amygdala-lesioned mice. The flight responses disappeared in the closed box, and retreat responses were most commonly observed both in the closed box and on the open platform in eight out of the 10 amygdala-lesioned mice. However, the remaining two mice (1,494^*^ and 1^*^) frequently showed flight responses in the closed box. The histological assessment revealed that the amygdala lesions, especially those in the basolateral (BL) and basomedial (BM) complex, were much smaller (– or +) in 1,494^*^ and 1^*^ compared with the other animals (Bottom two rows in Table 2). Therefore, these two animals were excluded from the analysis. The statistics in the remaining 8 mice with the amygdala lesion showed that the retreat responses were the most common both in the closed box and on the open platform as shown in Figure 3C2 (*n* = 8; ^*^: *p* < 0.05, ^**^ *p* < 0.01, Bonferroni's multiple comparison test). Reflecting such changes in the behavioral response pattern, the travel distance became short, and the peak velocity became slow and exhibited no significant difference between the closed box and the open platform as shown in Figure 4C2 (*n* = 8; *N.S.: p* > 0.05, Bonferroni's multiple comparison test). Furthermore, if we compare the sham-lesioned and amygdala-lesioned mice (Figures 3C1,C2), the frequency of flight responses was higher (*p* < 0.01, Bonferroni's multiple comparison test) and that of retreat responses was lower (*p* < 0.05, Bonferroni's multiple comparison test) in the amygdala-lesioned mice than in the sham-lesioned mice in the closed box. However, these differences were not found between these two groups on the open platform (*p* > 0.05, Bonferroni's multiple comparison test).

Table 2 shows the pseudoquantitative evaluation of the lesion extent in various subnuclei of the amygdala, CPu, Pir, En, and Hip, in the 10 mice administered with the ibotenic acid injections. 1,494^*^ and 1^*^ were excluded from the data as described above. These datasets suggested that even though the lesion was extended to the outside of the amygdala in some animals, the behavioral phenotype appeared to be most correlated with the lesion extent in BL and BM, which were the input stage of the amygdala complex.

## Discussion

In this study, we investigated the bilateral amygdala lesion on the environment dependency of the defense responses induced by the pathway-selective optogenetic activation of the uncrossed output pathway from the dSC in the mouse. Among the subnuclei of the amygdala, BL and BM appeared to be critical. It has been considered that the amygdala plays a critical role in the memory-dependent contextual modulation of the fear responses. In contrast, the present results showed that the amygdala is also critical in sensing the environmental setting and control of the defense behavior even at the first encounter of the fearful stimulus. This gives us a novel view of the role of the amygdala in behavioral regulation.

### Methodological Consideration

In this study, we used the double viral vector technique for the selective activation of the SC defense pathway as shown in our preceding study (Isa et al., 2020). Here, we injected the retrograde gene transfer vector carrying Cre into the CnF and anterograde vector AAV-EF1α-DIO-hChR2(H134R)-FP into the SC on the same side and expressed ChR2 in the uncrossed output pathway from the dSC. Here, because it was difficult to make amygdala lesions by insertion of microcannulas after we implanted the optic fibers for the optogenetic stimulations, we separated the animals with sham or the bilateral amygdala lesion before implantation of the optic fibers. Because of this experimental limitation, we could not use the same animals before and after the amygdala lesion, and instead, we had to separate the animals into two groups, that is, sham-lesioned and amygdala-lesioned animals. Therefore, careful considerations about the data sampling were needed.

To obtain a consistent dataset, we chose only the mice with successful expression of ChR2 and clear behavioral responses to the photostimulation in the sham-lesioned group, both in the closed box and on the open platform. We started the stimulation experiments 3 weeks after the injection of the second vector; however, if we could not observe the behavioral responses repeatedly, we waited for another 1 week to get more stable responses and obtained the dataset. At this stage, we found that no mice showed retreat responses on the open platform without exhibiting flight responses in the closed box. In the amygdala-lesioned group, the experimental arrangement did not allow us to check the existence of flight response in the closed box. Therefore, we first sampled the data from the 10 mice, which showed the robust retreat responses on the open platform for further analysis. However, we excluded 2 mice (1,494^*^ and 1^*^) from the dataset because the amygdala lesion was not sufficient as compared with the other eight mice as shown in Table 2. Actually, these mice showed flight responses frequently in the closed box. In the remaining eight mice, the flight responses mostly disappeared in the closed box and instead, the retreat responses were commonly observed. The current dataset clearly showed statistically significant changes in the behavioral response pattern, that is, the disappearance of the flight responses in the closed box (Figure 3C2) and corresponding shortening in the travel distance and slowing down the peak velocities (Figure 4C2).

### Technical Problems Related to the Extent of Amygdala Lesion

We experienced technical difficulty in making a selective lesion of the amygdala complex. We aimed at the basal and central nuclei of the amygdala; however, the lesion was not complete but sometimes extended to outside the amygdala. Therefore, we carefully examined the lesion extent in the sections with anti-NeuN immunostaining and made a pseudoquantitative estimation of the lesion extent in the individual subnuclei of the amygdala complex and other structures surrounding the amygdala such as CPu, Pir, En, and Hip as shown in Table 2. Then, it was found that although the lesion extended to other structures, the behavioral phenotype was most clearly correlated with the lesion extent in the BL and BM. Therefore, we concluded that the BL and BM underlie the environment dependency of the defense responses induced by the activation of the SC defense pathway.

### Possible Mechanism of Amygdala Involvement in Environment Dependency

Traditionally, the amygdala has been considered to play critical roles in the context-dependent modulation of fear responses. If a mouse receives a foot shock stimulus in a certain environment, it shows a freezing response on the next occasion if it is placed in the same environment. However, if the mouse is placed in an apparently different environment, it does not show freezing. If the amygdala is lesioned, the freezing response disappears even if the mouse is placed in the environment where it received the foot shock previously (Koo et al., 2004; Amano et al., 2011). Based on such observation, the involvement of the amygdala in the formation of fear memory has been established (Duvarci and Pare, 2014; Izquierdo et al., 2016).

**Figure 5:**
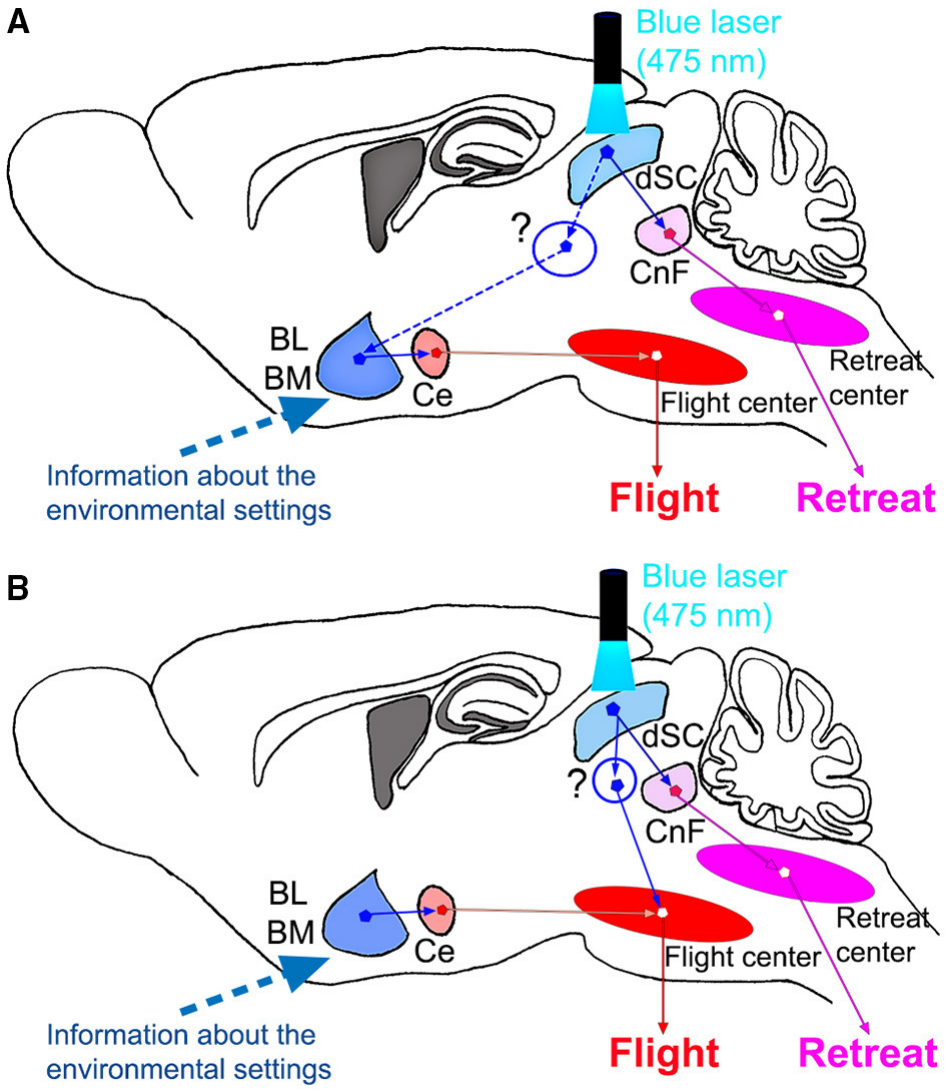
Proposed schematic diagrams of the circuit organization underlying the environment dependency of the defense responses. **(A)** Activation of flight response by dSC *via* the amygdala. **(B)** Amygdala modulates the flight center, which is activated directly by the dSC stimulation. CnF, cuneiform nucleus; dSC, deep layers of the superior colliculus.

In this study, however, it was found that contextual modulation of the behavioral responses of the mice to stimulation of the SC defense pathway was observed without preceding experience of the stimulation. From the first occasion of the photostimulation, the mice showed flight responses in the closed box and retreat responses on the open platform. This means that the mice understood the environment and made the decision of the appropriate defense responses immediately by integrating the information about the environmental setting and the visual information, which activates the SC defense pathway neurons, such as the predator approaching from above (Redgrave et al., 1986, 1987). The present results showed that after the amygdala lesion, the mice showed retreat both in the closed box and on the open platform. This suggests that the default response to the photostimulation is the retreat with backward locomotion and the amygdala is involved in forming the flight responses to the corner to immediately hide themselves, in addition to or by suppression of the retreat responses if the mice are placed in the closed environment. This may make sense because the CnF, the primary target of the SC defense pathway, has been considered to be the locomotor center or forming the central pattern generator for locomotion (Shik et al., 1966; Mori et al., 1978; Lee et al., 2014; van der Zouwen et al., 2021). Based on this assumption, we propose the circuit diagrams in Figure 5. This figure includes two possible mechanisms. As shown in Figure 5A, the SC defense pathway projects to the CnF and successively to the retreat center in the medial pontomedullary reticular formation (Isa et al., 2020). The CnF has been shown to regulate the reticulospinal neurons (Bretzner and Brownstone, 2013; Caggiano et al., 2018), which controls the backward locomotion for the retreat (Shimamura and Kogure, 1983; Zelenin, 2011). In addition, the SC defense pathway projects collaterals to various nuclei in the mesencephalon and thalamus which project to the amygdala, such as the periaqueductal gray matter, posterior thalamic nucleus triangular, zona incerta, and lateral hypothalamus (Doron and Ledoux, 2000; Kim et al., 2013; Evans et al., 2018; Branco and Redgrave, 2020; Isa et al., 2020; Ferreira-Pinto et al., 2021). And as shown in Figure 5A, the BL and/or BM integrate the information about the environmental setting (closed box in this case) and the visuomotor signals from the SC defense pathway and activate the downstream flight center through the central amygdaloid nucleus (Ce). Another possibility shown in Figure 5B suggests that the amygdala simply mediates the information about the environmental settings and integration with the visuomotor signals from the SC defense pathway takes place at the level of the flight center. To obtain a clue to conclude these two-contrasting hypotheses, we further need the information about how the environmental setting is represented in the brain, influences the activity of amygdala neurons, and affects the behaviors. For the amygdala to mediate the signals for a quick decision to make the flight responses to protect the mice from the predator, the amygdala should contain some representation of the environmental settings to make the decision. Since this seems to occur without any preceding experience, the representation should be implemented innately. A recent study in macaques showed that neurons in the amygdala encode “contexts” provided by the environment (Maeda et al., 2018), which may support this hypothesis. Comparison of the activity from the amygdala neurons preceding the photostimulation in the closed box and on the open platform would give us insights into the representation of the environmental settings. Furthermore, how such information about the environmental setting is integrated with the signal from the SC defense pathway neurons? Does the integration take place in the amygdala or the downstream flight center(s)? These questions should be addressed in future studies by using further circuit dissection techniques.

In this study, we mimicked the looming stimuli or approach of predators by the artificial optogenetic activation of the SC defense pathway. Therefore, it can be criticized that it is still unclear how the amygdala affects natural environment-dependent defensive responses. Because the environment dependency of the defense response we observed by the optogenetic stimulation was robust and mimic the natural behavior of rodents, we believe that this would surely be the case for the natural behaviors of the animals. However, the hypothesis should be tested in future in a more natural condition to induce the defense behavior.

## Data Availability Statement

The raw data supporting the conclusions of this article will be made available by the authors, without undue reservation.

## Ethics Statement

The animal study was reviewed and approved by the Institutional Animal Care and Use Committee of the Graduate School of Medicine, Kyoto University.

## Author Contributions

KI, KT, TS, and TI designed the experiments. KI, KT, SI, SK, and TS conducted the behavioral experiments and analyzed the data and conducted histological experiments and analyzed the data. KK designed and provided the viral vectors used in this study. All authors contributed to writing the manuscripts.

## Funding

This study was supported by AMED under Grant Number JP18dm0207020, a grant-in-aid for Scientific Research on Innovative Areas Adaptive Circuit Shift (Grant No. 26112003) and Hyper-adaptability (Grant No. 19H05723) from the MEXT Japan, KAKENHI Kiban (A) (Grant No. 19H01011) from the JSPS Japan, and JST/CREST (Grant ID: JPMJCR1651) to TI. SK was supported by the IBRO-APRC Exchange Fellowship Grant.

## Conflict of Interest

The authors declare that the research was conducted in the absence of any commercial or financial relationships that could be construed as a potential conflict of interest.

## Publisher's Note

All claims expressed in this article are solely those of the authors and do not necessarily represent those of their affiliated organizations, or those of the publisher, the editors and the reviewers. Any product that may be evaluated in this article, or claim that may be made by its manufacturer, is not guaranteed or endorsed by the publisher.

## Supplementary Material

Supplementary Video 1 Retreat response in a sham-lesioned mouse on the open platform. The period of optogenetic stimulation is indicated by a blue square in the video. Playback is real speed.

Supplementary Video 2 Flight response in a sham-lesioned mouse in the closed box. The period of optogenetic stimulation is indicated by a blue square in the video. Playback is real speed.

Supplementary Video 3 Retreat response in an amygdala-lesioned mouse on the open platform. The period of optogenetic stimulation is indicated by a blue square in the video. Playback is real speed.

Supplementary Video 4 Retreat response in an amygdala-lesioned mouse in the closed box. The period of optogenetic stimulation is indicatead by a blue square in the video. Playback is real speed.

## References

[B1] Amano T. Duvarci S. Popa D. Paré D. (2011). The fear circuit revisited: Contributions of the basal amygdala nuclei to conditioned fear. J. Neurosci. 31, 15481–15489. 10.1523/JNEUROSCI.3410-11.2011 22031894 PMC3221940

[B2] Bolles R. C.. (1970). Species-specific defense reactions and avoidance learning. Psychol. Rev. 77, 32–48. 10.1037/h00285896891452

[B3] Branco T. Redgrave P. (2020). The neural basis of escape behavior in vertebrates. Annu. Rev. Neurosci. 43, 417–439. 10.1146/annurev-neuro-100219-122527 32259462

[B4] Bretzner F. Brownstone R. M. (2013). Lhx3-Chx10 reticulospinal neurons in locomotor circuits. J. Neurosci. 33, 14681–14692. 10.1523/JNEUROSCI.5231-12.2013 24027269 PMC6705172

[B5] Caggiano V. Leiras R. Goñi-Erro H. Masini D. Bellardita C. Bouvier J.. (2018). Midbrain circuits that set locomotor speed and gait selection. Nature. 553, 455–460. 10.1038/nature25448 29342142 PMC5937258

[B6] Dean P. Redgrave P. Westby G. W. (1989). Event or emergency? Two response systems in the mammalian superior colliculus. Trends Neurosci. 12, 137–147. 10.1016/0166-2236(89)90052-0 2470171

[B7] Doron N. N. Ledoux J. E. (2000). Cells in the posterior thalamus project to both amygdala and temporal cortex: a quantitative retrograde double-labeling study in the rat. J. Comp. Neurol. 425, 257–274. 10.1002/1096-9861(20000918)425:2&lt;257::AID-CNE8&gt;3.0.CO;2-Y 10954844

[B8] Duvarci S. Pare D. (2014). Amygdala microcircuits controlling learned fear. Neuron. 82, 966–980. 10.1016/j.neuron.2014.04.042 24908482 PMC4103014

[B9] Evans D. A. Stempel A. V. Vale R. Ruehle S. Lefler Y. Branco T. (2018). A synaptic threshold mechanism for computing escape decision. Nature. 558, 590–594. 10.1038/s41586-018-0244-6 29925954 PMC6235113

[B10] Fanselow M. S. Lester L. S. (1988). A functional behavioristic approach to aversively motivated behavior: Predatory imminence as a determinant of the topography of defensive behavior. In Evolution and Learning. Bolles RC, Beecher MD (Eds). Earlbaum:Hillsdale NJ. p. 185–211.

[B11] Ferreira-Pinto M. J. Kanodia H. Falasconi A. Sigrist M. Esposito M. S. Arber S. (2021). Functional diversity for body actions in the mesencephalic locomotor region. Cell. 184, 1–15. 10.1016/j.cell.2021.07.002 34302739 PMC8382160

[B12] Franklin K. B. J. Paxinos G. (2008). The mouse brain. Third edition (New York: Academic Press).

[B13] Hanawa H. Hematti P. Keyvanfar K. Metzger M. E. Krouse A. Donahue R. E.. (2004). Efficient gene transfer into rhesus repopulating hematopoietic stem cells using a simianimmunodeficiency virus-based lentiviral vector system. Blood. 103, 4062–4069. 10.1182/blood-2004-01-0045 14976042

[B14] Hanawa H. Kelly P. F. Nathwani A. C. Persons D. A. Vandergriff J. A. Hargrove P.. (2002). Comparison of various envelop protein for their ability to pseudotype lentiviral vectors and transduce primitive hematopoietic cell from human blood. Mol. Ther. 5, 242–251. 10.1006/mthe.2002.0549 11863413

[B15] Isa K. Sooksawate T. Kobayashi K. Kobayashi K. Redgrave P. Isa T. (2020). Dissecting the tectal output channels for orienting and defense responses. eNeuro. 7:ENEURO.0271-20.2020. 10.1523/ENEURO.0271-20.2020 PMC7540932 32928881

[B16] Izquierdo I. Furini C. R. Myskiw J. C. (2016). Fear memory. Physiol. Rev. 96, 695–750. 10.1152/physrev.00018.2015 26983799

[B17] Janak P. H. Tye K. M. (2015). From circuits to behaviour in the amygdala. Nature. 517, 284–292. 10.1038/nature14188 25592533 PMC4565157

[B18] Johansen J. P. Cain C. K. Ostroff L. E. LeDoux J. E. (2011). Molecular mechanisms of fear learning and memory. Cell. 147, 509–524. 10.1016/j.cell.2011.10.009 22036561 PMC3215943

[B19] Kato S. Kobayashi K. Kobayashi K. (2014). Improved transduction efficiency of a lentiviral vector for neuron-specific retrograde gene transfer by optimizing the junction of fusion envelope glycoprotein. J. Neurosci. Methods. 227, 151–158. 10.1016/j.jneumeth.2014.02.015 24613797

[B20] Kim E. J. Horovitz O. Pellman B. A. Tan L. M. Li Q. Richter-Levin G.. (2013). Dorsal periaqueductal gray-amygdala pathway conveys both innate and learned fear responses in rats. Proc. Natl. Acad. Sci. USA. 110, 14795–14800. 10.1073/pnas.1310845110 23959880 PMC3767534

[B21] Kobayashi K. Sano H. Kato S. Kuroda K. Nakamuta S. Isa T.. (2016). Survival of corticostriatal neurons by Rho/Rho-kinase signaling pathway. Neurosci. Lett. 630, 45–52. 10.1016/j.neulet.2016.07.020 27424794

[B22] Koo J. W. Han J. S. Kim J. J. (2004). Selective neurotoxic lesions of basolateral and central nuclei of the amygdala produce differential effects on fear conditioning. J. Neurosci. 24, 7654–7662. 10.1523/JNEUROSCI.1644-04.2004 15342732 PMC6729625

[B23] LeDoux J. E. Iwata J. Cicchetti P. Reis D. J. (1988). Different projections of the central amygdaloid nucleus mediate autonomic and behavioral correlates of conditioned fear. J. Neurosci. 8, 2517–2529. 10.1523/JNEUROSCI.08-07-02517.1988 2854842 PMC6569498

[B24] Lee A. M. Hoy J. L. Bonci A. Wilbrecht L. Stryker M. P. Niell C. M. (2014). Identification of a brainstem circuit regulating visual cortical state in parallel with locomotion. Neuron. 83, 455–466. 10.1016/j.neuron.2014.06.031 25033185 PMC4151326

[B25] Maeda K. Kunimatsu J. Hikosaka O. (2018). Amygdala activity for the modulation of goal-directed behavior in emotional contexts. PLoS Biol. 16, e2005339. 10.1371/journal.pbio.2005339 PMC5988268 29870524

[B26] Mori S. Nishimura H. Kurakami C. Yamamura T. Aoki M. (1978). Controlled locomotion in the mesencephalic cat: distribution of facilitatory and inhibitory regions within pontine tegmentum. J. Neurophysiol. 41, 1580–1591. 10.1152/jn.1978.41.6.1580 731291

[B27] Redgrave P. Mitchell I. J. Dean P. (1987). Further evidence for segregated output channels from superior colliculus in rat: ipsilateral tecto-pontine and tecto-cuneiform projections have different cells of origin. Brain Res. 413, 170–174. 10.1016/0006-8993(87)90165-X 3594255

[B28] Redgrave P. Odekunle A. Dean P. (1986). Tectal cells of origin of predorsal bundle in rat: location and segregation from ipsilateral descending pathway. Exp. Brain Res. 63, 279–293. 10.1007/BF00236845 3093259

[B29] Sahibzada N. Dean P. Redgrave P. (1986). Movements resembling orientation or avoidance elicited by electrical stimulation of the superior colliculus in rats. J. Neurosci. 6:723–733. 10.1523/JNEUROSCI.06-03-00723.1986 3958791 PMC6568468

[B30] Shik M. L. Orlovskii G. N. Severin F. V. (1966). Organization of locomotor synergism. Biofizika. 11, 879–886. 6000596

[B31] Shimamura M. Kogure I. (1983). Discharge patterns of reticulospinal neurons corresponding with quadrupedal leg movements in thalamic cats. Brain Res. 260, 27–34. 10.1016/0006-8993(83)90761-8 6824953

[B32] Sooksawate T. Isa K. Matsui R. Kato S. Kinoshita M. Kobayashi K.. (2013). Viral vector-mediated selective and reversible blockade of the pathway for visual orienting in mice. Front. Neural Circuits. 7, 162. 10.3389/fncir.2013.00162 PMC3795302 24130520

[B33] Terburg D. Scheggia D. Triana Del Rio R. Klumpers F. Ciobanu A. C. Morgan B.. (2018). The basolateral amygdala is essential for rapid escape: a human and rodent study. Cell. 175, 723–735.e16. 10.1016/j.cell.2018.09.028 30340041 PMC6198024

[B34] van der Zouwen C. I. Boutin J. Fougère M. Flaive A. Vivancos M. Santuz A.. (2021). Freely behaving mice can brake and turn during optogenetic stimulation of the mesencephalic locomotor region. Front. Neural Circuits. 15, 639900. 10.3389/fncir.2021.639900 PMC8062873 33897379

[B35] Zelenin P. V.. (2011). Reticulospinal neurons controlling forward and backward swimming in the lamprey. J. Neurophysiol. 105, 1361–1371. 10.1152/jn.00887.2010 21248057

